# Development of insect life tables: comparison of two demographic methods of *Delia antiqua* (Diptera: Anthomyiidae) on different hosts

**DOI:** 10.1038/s41598-017-05041-5

**Published:** 2017-07-06

**Authors:** Shuoying Ning, Wenchao Zhang, Yan Sun, Jinian Feng

**Affiliations:** Key Laboratory of Plant Protection Resources and Pest Management of the Ministry of Education, College of Plant Protection, Northwest A&F University, Yangling, Shaanxi 712100 P. R. China

## Abstract

In this study, we first construct an age-stage, two-sex life table for onion maggot, *Delia antiqua*, grown on three host plants: onion, scallion, and garlic. We found that onion is the optimal host for this species and populations grown on onion have maximum fecundity, longest adult longevity and reproduction period, and the shortest immature developmental time. In contrast, the fecundity on other hosts was lower, particularly on garlic, but these crops can also serve as important secondary hosts for this pest. These data will be useful to the growers to develop specific integrated management programs for each of hosts. We also compared the demographic analyses of using individually-reared and group-reared methods. These two methods provided similar accurate outcomes for estimating insect population dynamics for this species. However, for gregarious species, using the individually-reared method to construct insect life tables produces inaccurate results, and researchers must use group-reared method for life table calculations. When studying large groups of insect, group-reared demographic analysis for age-stage, two-sex life table can also simplify statistical analysis, save considerable labor, and reduce experimental errors.

## Introduction

Onion maggot, *Delia antiqua* (Meigen) (Diptera: Anthomyiidae), is one of the most serious subterranean pest of scallion (*Allium fistulosum* L*)*, garlic (*Allium sativem* L) and onion (*Allium cepa* L*)* in China as well as in many regions throughout the world^1–3^, and yields can be significantly reduced by larval attacks^4, 5^. The effect on the development and stability of these insects are the results of species and the quality of host plants^6, 7^. Host plants can obviously affected the population dynamics of divergent residents^8^. Therefore, it is crucial to thoroughly understand the ecology of a pest on different hosts from an agricultural production perspective and to formulate an integrated pest management program.

Life tables are a fundamental tool used to study population ecology^9, 10^. Life tables summarize the survival and reproductive potential of insect populations on different hosts, and under various environmental conditions^11–13^. As introduced by Chi^14, 15^, the age-stage, two-sex life table represents an improvement over traditional life table because it considers males and can describe stage differentiation^16^. Furthermore, age-stage, two-sex life table can precisely reveal the actual life history of the insect species and have been widely applied to study various ecological aspects of insect pests and their natural enemies^17, 18^. However, for some species such as *Bactrocera dorsalis* (Hendel) (Diptera: Tephritidae) and *Bradaysia odoriphaga* (Yang et Zhang) (Diptera: Sciaridae), the larvae are gregarious and may have higher mortality rates if they are reared individually^19, 20^. For these types of gregarious species, larvae must be reared in groups in order to produce an accurate life table.

This is the first study to compare the population dynamics including development, survival rate and fecundity of the onion maggot reared on three major *Allium* hosts. We measured these parameters on onion, scallion and garlic and analyzed them using an age-stage, two-sex life table. Furthermore, we compared the demographics of the individually-reared and group-reared methods on each of these host plants using an age-stage two-sex life table. These data will be useful in formulating an integrated control strategy for *D. antiqua* and the group-reared method will provide a simple and convenient way to construct life tables for insect population ecology research.

## Results

### Individually reared method

Values for the developmental time of each stage, longevity of males and females, adults pre-oviposition (APOP), total pre-oviposition (TPOP), female fecundity, and female oviposition days are listed in Table 1. The developmental time of eggs (*P*_OS_ = 0.5478; *P*_SG_ = 0.0008; *P*_OG_ < 0.0001) and larvae (*P*_OS_ = 0.9011; *P*_SG_ < 0.0001; *P*_OG_ < 0.0001) on garlic were significantly longer than on onion and scallion, but the pupal stage on garlic (*P*_OS_ = 0.9391; *P*_SG_ < 0.0001; *P*_OG_ < 0.0001) was significantly shorter than on others two hosts. The male (*P*_OS_ = 0.3620; *P*_SG_ < 0.0001; *P*_OG_ < 0.0001) and female longevity (*P*_OS_ = 0.8870; *P*_SG_ < 0.0001; *P*_OG_ < 0.0001), and oviposition days (*P*_OS_ = 0.3417; *P*_SG_ < 0.0001; *P*_OG_ < 0.0001) on garlic were significantly shorter than reared on onion and scallion. There were significant differences between the adult pre-oviposition (APOP) (*P*_OS_ < 0.0001; *P*_SG_ < 0.0001; *P*_OG_ < 0.0001), total pre-oviposition (TPOP) (*P*_OS_ = 0.0437; *P*_SG_ < 0.0001; *P*_OG_ < 0.0001), and female fecundity (*P*_OS_ < 0.0001; *P*_SG_ = 0.0006; *P*_OG_ < 0.0001) reared on different host plants. The mean pre-oviposition period when reared on onion, scallion and garlic were 6.5d, 7.56d and 8.53d, respectively. When reared on garlic, the first female adult emerged somewhat later (day 33) than other hosts. The mean TPOP on garlic was 44.13d, significantly later than on onion (39.55d) and scallion (40.28d). The mean fecundity of females (350.45 eggs) was significantly higher on onion, followed by scallion (303.28 eggs) and significantly lower on garlic (267.01 eggs).

**Table 1:** Developmental time, adult longevity, fecundity, adult preoviposition period (APOP), total preoviposition period (TPOP), and oviposition days of *Delia antiqua* on different host plants of Individually reared method.

Parameters	Stage	Host plant		
		Onion	Scallion	Garlic
Developmental time (days)	Egg	3.20 ± 0.06^a^	3.26 ± 0.06^a^	3.67 ± 0.11^b^
	Larva	15.37 ± 0.09^a^	15.35 ± 0.08^a^	16.7 ± 0.10^b^
	Pupa	14.37 ± 0.12^a^	14.36 ± 0.07^a^	14.22 ± 0.07^b^
Adult longevity (days)	Male	49.77 ± 0.58^a^	50.52 ± 0.58^a^	47.42 ± 0.70^b^
	Female	59.95 ± 0.66^a^	59.83 ± 0.57^a^	56.27 ± 1.19^b^
APOP (days)		6.50 ± 0.11^a^	7.56 ± 0.12^b^	8.53 ± 0.13^c^
TPOP (days)		39.55 ± 0.25^a^	40.28 ± 0.26^b^	44.13 ± 0.51^c^
Fecundity (eggs)		350.45 ± 2.91^a^	303.28 ± 4.78^b^	267.01 ± 8.64^c^
Oviposition days		20.3 ± 0.52^a^	19.56 ± 0.61^a^	14.13 ± 0.74^b^

The difference between treatments were evaluated by using paired bootstrap test. Values are means ± standard errors; ^a,b,c^Means in a row followed by different letters are significantly different at *p* < 0.05 by using paired bootstrap test (Table S1).

**Figure 1:**
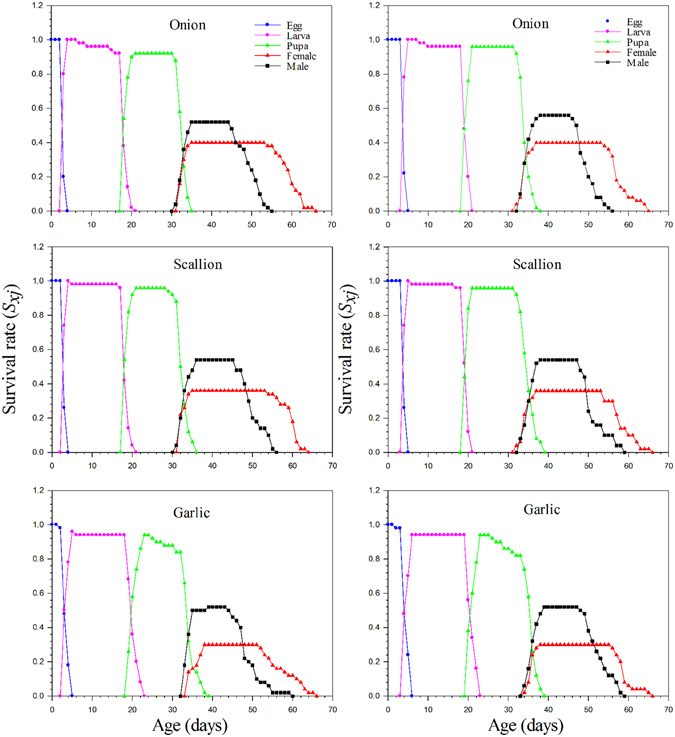
Survival rate to different development stages of *Delia antiqua* on different host plants. Left: Individually reared method; Right: Group reared method.

The age-stage specific survival rate (*s*_*xj*_) of *Delia antiqua* on different host plants indicates the probability that a new born will survival to age *x* and develop to stage *j* (Fig. 1). Considering the variable developmental rate among individuals, there are significant overlaps between stages in the survival curves. The total developmental time was not significantly different between onion and scallion. The developmental time was longer and survival rate was lower on garlic. The first females and males emerged on 34D and 33D, respectively. The survival rates of eggs and larvae were all approximately to 96% on different host plants. The survival rate of pupae on garlic was 8% lower than on the other host plants.

**Figure 2:**
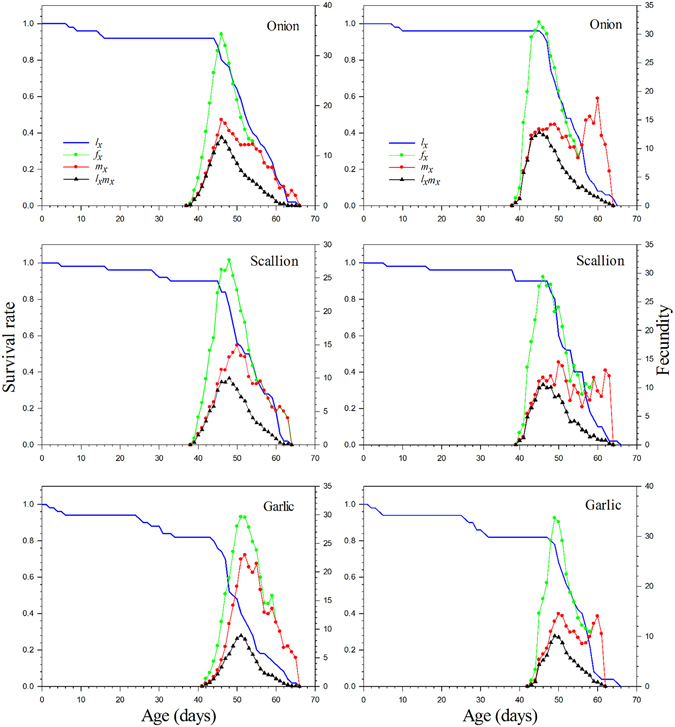
The age-specific survival rate (*l*_*x*_), female age-specific fecundity (*f*_*x*_), age-specific fecundity (*m*_*x*_), and age-specific maternity (*l*_*x*_ *m*_*x*_) versus age of *Delia antiqua* on different host plants. Left: Individually reared method; Right: Group reared method.

By ignoring the stage differentiation, a single age-stage survival rate (*l*_*x*_) gives the probability that an egg will survive to age *x* (Fig. 2). On onion and scallion, the *l*_*x*_ curved dropped only slightly during the early stages, indicating that the mortality rate at this stage was low. The age-stage specific fecundity (*f*_*x*_) curve peak on onion was higher than those on scallion and garlic. The curve of age specific fecundity (*m*_*x*_) showed that reproduction began at age 38D and 39D respectively on onion and scallion. However, the first reproduction on garlic occurred later at age 42D, and the fecundity on garlic was significantly lower than on the other host plants.

The mean of net reproductive rate (*R*_*0*_) of the *Delia antiqua* reared on all three hosts were significantly different and the value was highest on onion (140.18), followed by scallion (109.18) and garlic (80.10) (*P*_OS_ < 0.0001; *P*_SG_ < 0.0001; *P*_OG_ < 0.0001) (Table 2). The generation time (*T*) was significantly lower on onion, followed by scallion, and was longest on garlic (*P*_OS_ = 0.0136; *P*_SG_ < 0.0001; *P*_OG_ < 0.0001). The average intrinsic rate of increase (*r*) (*P*_OS_ = 0.2126; *P*_SG_ = 0.0592; *P*_OG_ = 0.0003) and finite rate of increase (*λ*) (*P*_OS_ = 0.2159; *P*_SG_ = 0.0588; *P*_OG_ = 0.0032) was significantly higher on onion than on garlic.

**Table 2:** Net reproductive rate (*R*_*0*_), the intrinsic rate of increase (*r*), finite rate of increase (*λ*), and generation time (*T*) of *Delia antiqua* on different host plant of individually reared method.

Parameters	Host plant		
	Onion	Scallion	Garlic
*R*_*0*_	140.18 ± 14.24^a^	109.18 ± 10.69^b^	80.10 ± 7.51^c^
*r*	0.1027 ± 0.0038^a^	0.0960 ± 0.0045^ab^	0.0846 ± 0.0045^b^
*λ*	1.1082 ± 0.0042^a^	1.1007 ± 0.0045^ab^	1.0882 ± 0.0049^b^
*T*	48.12 ± 0.23^a^	48.91 ± 0.22^b^	51.84 ± 0.61^c^

The data of treatments were compared by using paired bootstrap test. Values are means ± standard errors; ^a,b,c^Means in a row followed by different letters are significantly different at *p* < 0.05 by using paired bootstrap test (Table S2).

**Figure 3:**
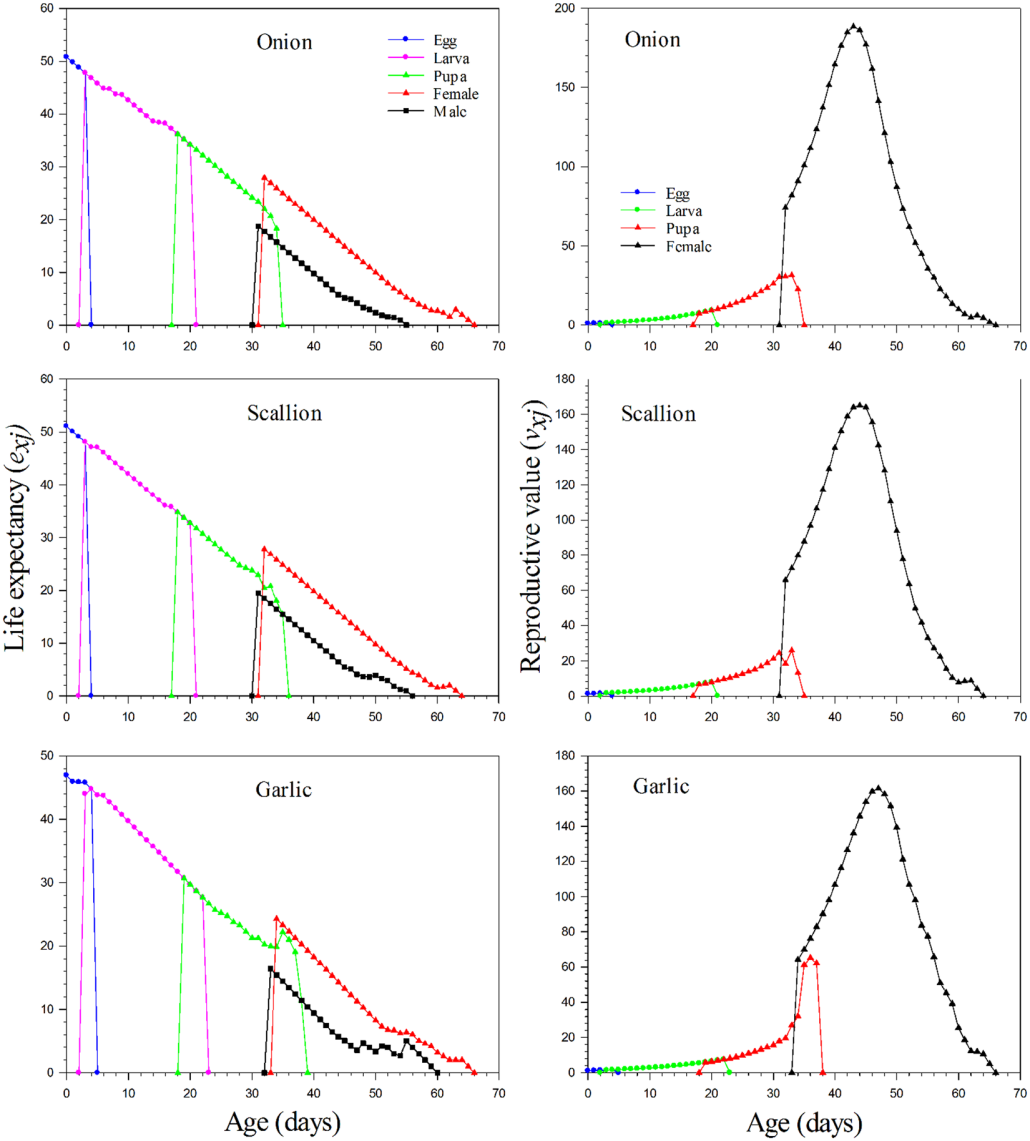
The age-stage life expectancy and reproductive value of *Delia antiqua* on different host plants of individually reared method. Left: The age-stage life expectancy; Right: The age-stage reproductive value.

The age-stage life expectancy (*e*_*xj*_) is used to estimate the time an individual of age *x* and stage *j* is expected to live. The longevity of the *Delia antiqua* at age zero (*e*_*01*_) was 46.92d on garlic, which did differ from the longevity of 50.8d and 51.1d on onion and scallion, respectively (Fig. 3).

The age-stage reproductive value (*v*_*xj*_) shows the contribution of an individual from age *x* to stage *j* to the future population, and the reproductive values (*v*_*01*_) is exactly the same as the finite rate. The curves for reproductive value significantly increase when reproduction begins. As shown in Fig. 3, the reproductive values on different host plants at age zero, i.e., 1.1082d^−1^ on onion, 1.1007d^−1^ on scallion, and 1.0882d^−1^ on garlic, were not substantially different among the three hosts. The value of *v*_*xj*_ on onion and scallion increased to 74.15d^−1^ and 65.91d^−1^ respectively on 32D when females emerged; when reared on garlic, the *v*_*xj*_ value increased to 64.28d^−1^ on 34D when female emerged.

### Grouped reared method

The population parameters for group reared data calculated by using the age-stage, two-sex life tables for *Delia antiqua* on different host plants are shown in Table 3. Statistical differences among the population parameters of the *Delia antiqua* reared on the three hosts were similar to those previously reported from individually reared method. Again, the mean of net reproductive rate (*R*_*0*_) on insects reared on onion was significantly higher (143.02) than on scallion (114.56) and garlic (86.54) (*P*_OS_ = 0.0040; *P*_SG_ = 0.0033; *P*_OG_ = 0.0072). The generation time (*T*) was statistically the lowest on onion, followed by scallion and garlic (*P*_OS_ = 0.0003; *P*_SG_ < 0.0001; *P*_OG_ < 0.0001). The mean of the intrinsic rate of increase (*r*) (*P*_OS_ = 0.2316; *P*_SG_ = 0.0976; *P*_OG_ = 0.0077) and finite rate of increase (*λ*) (*P*_OS_ = 0.2316; *P*_SG_ = 0.0972; *P*_OG_ = 0.0075) were significantly lower for insects reared on garlic (0.0872d^−1^ and 1.0911d^−1^) than those reared on onion.

**Table 3:** Net reproductive rate (*R*_*0*_), the intrinsic rate of increase (*r*), finite rate of increase (*λ*), and generation time (*T*) of *D. antiqua* on different host plants of group reared method.

Parameters	Host plant		
	Onion	Scallion	Garlic
*R*_*0*_	143.02 ± 15.11^a^	114.56 ± 11.92^b^	86.54 ± 8.89^c^
*r*	0.1037 ± 0.0038^a^	0.0972 ± 0.0041^ab^	0.0872 ± 0.0045^b^
*λ*	1.1093 ± 0.0043^a^	1.1021 ± 0.0045^ab^	1.0911 ± 0.0048^b^
*T*	47.85 ± 0.17^a^	48.78 ± 0.16^b^	51.15 ± 0.11^c^

The data of treatments were compared by using paired bootstrap test. Values are means ± standard errors; ^a,b,c^Means in a row followed by different letters are significantly different at *p* < 0.05 by using paired bootstrap test (Table S3).

In the analyses of age-stage-sex population structures, the survival rate (*s*_*xj*_) was recorded daily at different developmental periods throughout the entire lifespan, with sex determined at the adult stage (Fig. 1). The total developmental time was not substantially different on onion and scallion, but on garlic it was the slowest. The survival rate of preadult individuals was 10% lower on garlic than on the other hosts.

The age-specific survival rate (*l*_*x*_), the female age-stage specific fecundity (*f*_*x*_), the age-specific fecundity (*m*_*x*_), and the age-specific maternity (*l*_*x*_ *m*_*x*_) of *Delia antiqua* were also calculated for the group-reared method in Fig. 2. At the early stages of development for insects reared on onion and scallion, the survival rate (*l*_*x*_) curve dropped more gradually than it did on garlic, indicating that the mortality rate at this stage was low on these two hosts. The curve of age specific fecundity (*m*_*x*_) showed that reproduction began at different ages on various hosts, i.e., 39D on onion, 40D on scallion, and 43D on garlic, and the fecundity on garlic was significantly less than on other host plants (*P*_OS_ = 0.0011; *P*_SG_ = 0.0021; *P*_OG_ < 0.0001).

**Figure 4:**
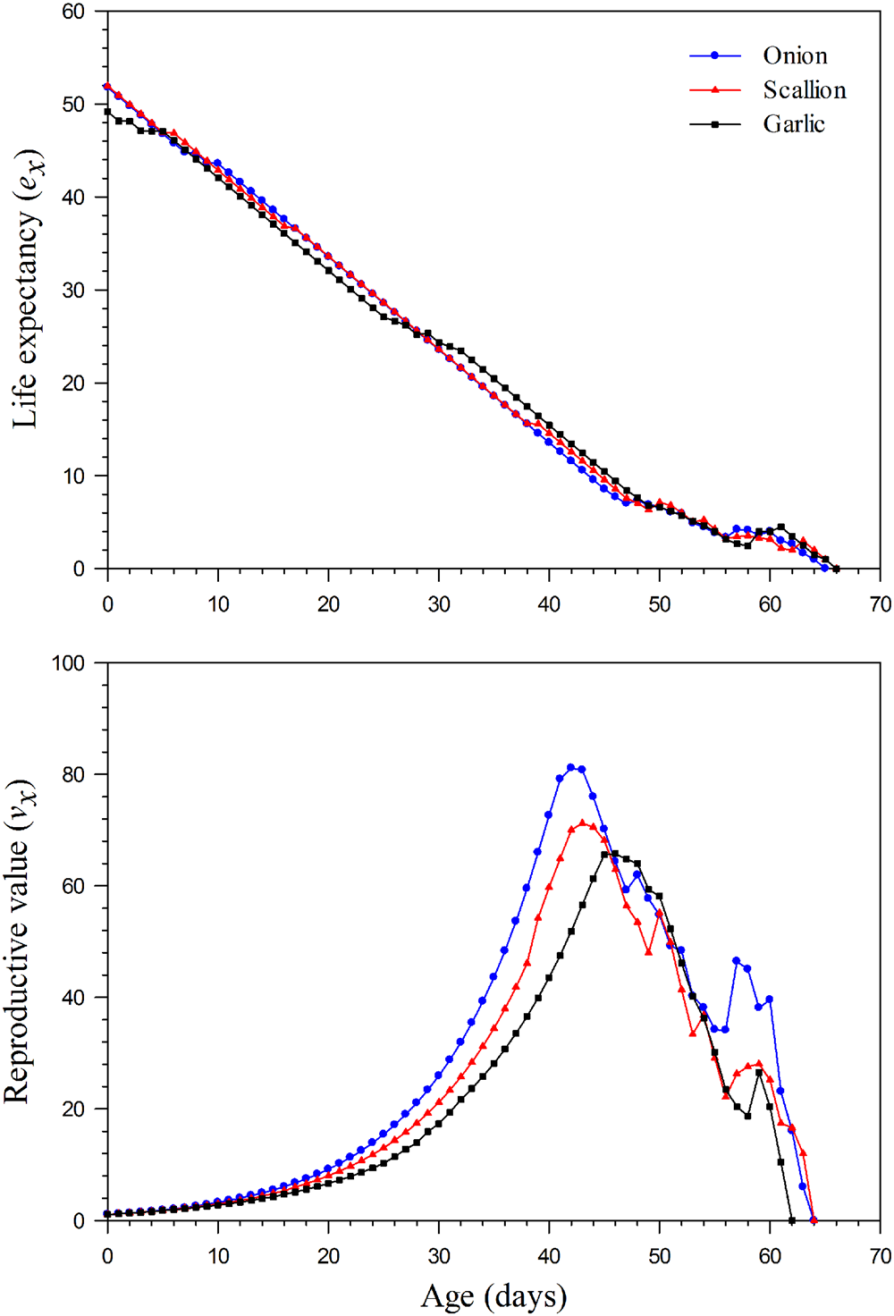
The age-specific life expectancy and reproductive value of *Delia antiqua* on different host plants of group reared method. Upper: The age-specific life expectancy; Below: The age-specific reproductive value.

The age-specific life expectancy (*e*_*x*_) estimates the time individuals of age *x* are expected to live (Fig. 4) and the age-specific reproductive value (*v*_*x*_) indicates the contribution of individuals from age *x* to the future population (Fig. 4). The longevity of the *Delia antiqua* at age zero (*e*_*0*_) was shorter on garlic (49.16d) than on onion (51.78d) and scallion (51.92d), respectively. When reproduction begins, the curves for reproductive value rapidly increase and the reproductive values at age zero on different host plants were almost consistent, i.e., 1.1093d^−1^ on onion, 1.1021d^−1^ on scallion, and 1.0911d^−1^ on garlic. The value of *v*_*x*_ on onion, scallion, and garlic increased to 31.93d^−1^, 25.74 d^−1^ and 25.82d^−1^ respectively when females began emerging. The peak of the *v*_*x*_ curve was higher on onion (81.10d^−1^ on 42D), followed by on scallion (71.21d^−1^ on 43D) and on garlic (65.75d^−1^ on 46D).

## Discussion

Onion maggot, *Delia antiqua*, is a serious chronic pest in subtropical regions throughout the world^1, 29^. Previous research has demonstrated that the main host of *Delia antiqua* is onion in most countries^29^. However, control of *Delia antiqua* has been a major problem on several species of the genus *Allium* in China and the population dynamics and damage periods vary among these different hosts^30^.

Results from this study support most previous work that has shown that different host plants can significantly affect the development, survival, and reproduction of insects^11, 16, 31^. This is the first study to use a life table approach to compare the population dynamics of onion maggot on all three of its major *Allium* hosts. Our work demonstrated that different host plants can affected the population growth rate of *Delia antiqua*, because of the differences in the immature developmental time, fecundity, and oviposition period. Onion maggot adults reared on onion lived longer and had maximum fecundity and reproduction periods, and the shortest immature developmental time (Table 1). Therefore, the onion can be regarded as an optimal host because onion maggot populations will build up more rapidly on onion than on other two *Allium* species examined. Although females reared on scallion and garlic have lower fecundity, these hosts are also suitable for the development of this pest and can serve as alternative hosts (Fig. 2). Because the age-stage, two-sex life table takes the variable developmental rate among individuals into consideration, the overlapping curves of survival rate (*s*_*xj*_) can be observed in (Fig. 1) and the age specific survival rate (*l*_*x*_ *)* curve can simply and correctly describe the changes in survival rates (Fig. 2). However, if a traditional female life table such as the Lewis-Leslie matrix was used for data analysis^32, 33^, the stage differentiations would not be observed^14^, and the *l*_*x*_ curve would not be accurate^16^.

Life table parameters are a useful method to assess population development, survival, and reproduction^9^. In our study, the intrinsic and finite rates of increase, and net reproductive rate were higher and mean generation time was shorter on onion compared with the other host plants tested (Table 2). These trends may be attributed to differences in nutrition and moisture content of host plants^30, 34^. Chi^14^ showed the relationship between the net reproductive rate (*R*_*0*_) and mean female fecundity (*F*) as *R*_*0*_ = *F* × (*N*_*f*_/*N*), where *N* is the total number of eggs used at the beginning of the life table study, and *N*_*f*_ is the number of female adults that emerged. In this study, the values of *R*_*0*_ and *F* on each host plant are consistent with this relationship and this formula can be used to detect errors in life table analysis^16^.

This study determined that *Delia antiqua* can survive, on average, longer than two months on all three host plants, and successfully produce offspring for at least a month. Because all of these *Allium* species can be considered as important economic hosts, we suggest that the information provided in this study comparing population demographics of the onion maggot can be used to develop specific integrated management programs for each of these primary hosts.

Population density significantly affects many aspects of insect activities, such as larvae growth^35^, adult fecundity^36^, egg mortality rate^37^, copulation^38^, and flight activity^39^, etc. As previously described above, the age-stage, two-sex life table has certain advantages for application in the study of insect population ecology compared to traditional life table^14–17, 40^. For some gregarious insects, larvae tend to aggregate in the field^16, 19, 20^, and if larvae are reared alone, they grow more slowly^41^ and have higher mortality than when reared together^19^.

In our study, we compared the demographic analysis of individually-reared and group-reared method on each of these three host plants using age-stage two-sex life table. The results show that the survival rate to different development stages and population parameters under these two types of demographic analysis on each host plant were consistent. Although the individual immature developmental time, adult longevity, adult preoviposition period, and total preoviposition period couldn’t be obtained from group-reared data because the mean of all individuals were calculated as a group, the survival rate of each stage and fecundity of the group reared method were similar to those of individually reared method. Because of the different calculation methods, the *m*_*x*_ and *f*_*x*_ curves reveal the variability of fecundity. However, if we considered the age specific survival rate (*l*_*x*_), the age-specific maternity curve will be similar^16^. In addition, the daily number of eggs laid by each female were used to assign the mean fecundity to each female and therefore did not affect *l*_*x*_ and *m*_*x*_ because Chi^14^ proved the relationship between *R*_*0*_ and *F*. Compared with the age-stage life expectancy (*e*_*xj*_), the age-specific life expectancy (*e*_*x*_) ignores the stage and estimates the time individuals of age *x* are expected to live. In addition, the age-specific reproductive value (*v*_*x*_) indicates the contribution of individuals from age *x* to the future population and ignores the stage as same as *e*_*x*_. As shown by our results, *e*_*0*_ and *e*_*01*_ were the mean longevity of all individuals and *v*_*01*_ and *v*_*0*_ were the same as the finite rate.

In conclusion, the two-sex life table is more accurate than traditional life table because it describes stage differentiation and includes both sexes. However, for some gregarious species, rearing larvae separately is not possible because it will produce inaccurate results. Under these circumstances, two-sex life table can utilize the demographic analysis of group-reared method to solve this problem. Then, if researchers focus on a very large groups of insects, demographic analysis of group reared data will provide a simple and convenient method to reduce the experimental error and simplify the statistical analysis of the data.

## Materials and Methods

### Plants

The three species of *Allium* were used in this study, i.e., Onion (*Allium cepa* L., variety ‘Xi’an Red’), garlic (*Allium sativum* L., variety ‘Xingping White’), and scallion (*Allium fistulosum* L., variety ‘Shaanxi Rock’). Plants were grown in a greenhouse at Northwest A&F University, Yangling, China and were maintained under identical conditions for colony maintenance (25 ± 1 °C, 60 ± 10% RH, photoperiod of 14:10 [L:D] h). No insecticides were applied to the plants. These plants were used as food for *D. antiqua* larvae and as an oviposition lure.

### Insect

Colonies of onion maggot were obtained from Key Laboratory of Plant Protection Resources and Pest Management of the Ministry of Education, located at Northwest A&F University, Yangling, China, where flies have been continuously reared for over 50 generations. Eggs, larvae and adults described below were maintained in an environmental chamber (21 °C, 50% RH, and a photoperiod of 16:8 [L:D] h). Larvae were reared on host plants in a petri dish covered by light blocking box to simulate the underground environment^21^. Adults were maintained in a mesh cage (40 × 40 × 30 cm) and were provisioned ad libitum with water and a diet of brewer’s yeast, powdered milk, sugar and soy peptone^22^. Flies were allowed to oviposit in a uncovered dish containing sand and peeled plant parts; eggs then were floated out of the sand using water^23^. Cohorts of similar age eggs were collected for laboratory experiments.

### Experiments

Fifty eggs within a 12 h period were collected from each of the three host plants respectively (Onion (*Allium cepa* L., variety ‘Xi’an Red’), garlic (*Allium sativum* L., variety ‘Xingping White’), and scallion (*Allium fistulosum* L., variety ‘Shaanxi Rock’). Because of the difficulty in identifying specific larval instars, we grouped all larval instars together as the larval stage.

#### Individually reared method

Fifty eggs were deposited singly, in vials. The larvae were fed fresh food daily^24^, and reared under the same conditions as previously described for eggs until pupation. Data were taken daily, to monitor egg hatched and the duration of each developmental stage of *Delia antiqua* on the three host plants. After the emergence of adults, we paired one female with one male and kept them in a mating cage and recorded the number of eggs daily until the adults died.

#### Group reared method

Fifty eggs were collected in group in a petri dish and the larvae were bred gregariously in these groups from hatching until pupation. Petri dishes were examined daily, and the number of eggs hatched, larvae, pupae, and adults were examined and recorded. After adult emergence, female and male adults were all confined together in a single mating cage and the number of eggs laid collectively and dead adults of both sexes were recorded daily until adults were all dead.

### Demographic analysis

#### Individually reared method

Raw data of daily development and reproduction of each individual were analyzed according to the age-stage, two-sex life table theory as described by Chi^14, 15^. The population parameters calculated were age-stage specific survival rate (*S*_*xj*_: the probability that a newly laid egg will survive to age *x* and stage *j*), age-stage specific fecundity (*f*_*xj*_: the mean fecundity of females at age *x*), age-specific survival rate (*l*_*x*_: the probability that a newly laid egg survives to age *x*), and age-specific fecundity (*m*_*x*_: the mean fecundity of individuals at age *x*).

In the age-stage, two-sex life table, *l*_*x*_ and *m*_*x*_ are calculated as^15^:

1 $${l}_{x}=\sum _{j=1}^{k}{s}_{xj}$$

2 $${m}_{x}=\frac{\sum _{j=1}^{k}{s}_{xj}{f}_{xj}}{\sum _{j=1}^{k}{s}_{xj}}$$

where *k* is the last stage of the study cohort.

The net reproductive rate (*R*_*0*_) represents the total number of offspring that an individual can produce during its lifetime and is calculated as^15^:

3 $${R}_{0}=\sum _{x=0}^{\infty }{l}_{x}{m}_{x}$$

The intrinsic rate of increase (*r*) is estimated by using the iterative bisection method from the Euler-Lotka equation with age indexed from zero as follows^25^:

4 $$\sum _{x=0}^{\infty }{e}^{-r(x+1)}{l}_{x}{m}_{x}=1$$

The finite rate of increase (*λ*) is calculated as follows^25^:

5 $$\lambda ={e}^{r}$$

The mean generation time (*T*) is defined as the length of time that a population requires to increase to the *R*_*0*_-fold of its size at the stable age-stage distribution, and is calculated as follows^25^:

6 $$T=\frac{\mathrm{ln}\,{R}_{0}}{r}$$

Age-stage life expectancy (*e*_*xj*_), i.e. the time that an individual of age *x* and stage *j* is expected to live, was calculated according to the method described by Chi and Su^17^ as:

7 $${e}_{xj}=\sum _{i=x}^{n}\sum _{j=y}^{m}{s^{\prime} }_{xj}$$

where *n* is the number of age groups and *m* is the number of stages, and *s*′_*ij*_ is the probability that an individual of age *x* and stage *j* will survive to age *i* and stage *y*. The age-stage reproductive value (*v*_*xj*_) was defined as the contribution of individuals of age *x* and stage *j* to the future population^12, 26^. In the age- stage, two-sex life table, it is calculated as^26^:

8 $${V}_{xj}=\frac{{e}^{-r(x+1)}}{{s}_{xj}}\sum _{i=x}^{n}{e}^{-r(x+1)}\sum _{j=y}^{m}{s^{\prime} }_{ij}{f}_{ij}$$

The means and standard errors of the life table parameters were estimated by using the bootstrap procedure with bootstrap number m = 40,000 to ensure precise estimates^27^. TWOSEX-MSChart^28^ for windows was used to analyze our age-stage two-sex life table data. The paired bootstrap test^11, 27^ was used to compare the differences in developmental time, adult longevity, adult preoviposition period (APOP), total preoviposition period (TPOP), oviposition days, and fecundity between treatments. The population parameters among the three treatment were also compared by using the paired bootstrap test based on the confidence interval of difference^11, 27^ where the *P* value of paired bootstrap test were defined as: *P*_OS_, Onion to Scallion; *P*_SG_, Scallion to Garlic; *P*_OG_, Onion to Garlic; the same below.

#### Group reared method

According to the age-stage two-sex life table theory^14, 15^, all of the data collected from different developmental stages and sexes were analyzed using TWOSEX-MSChart^28^. Because the insects were reared in groups, the number of individuals that survived to age *x* for each stage were recorded. The survival rate (*S*_*xj*_) to each age-stage unit is calculated as^20^:

9 $${s}_{xj}=\frac{{n}_{xj}}{{n}_{01}}$$

where *n*_*01*_ is the number of eggs used at the beginning of life table study and *n*_*xj*_ is the number of insects that survived to age *x* and stage *j*. Because we recorded the total number of eggs (*E*_*x*_) laid by all female adults (the fourth life stage) at age *x*, female age-specific fecundity *f*_*x4*_ is calculated as^20^:

10 $${f}_{x4}=\frac{{E}_{x}}{{n}_{x4}}$$

and the net reproductive rate is calculated as^15^:

11 $${R}_{0}=\sum _{x=0}^{\infty }\sum _{j=1}^{m}{s}_{xj}{f}_{xj}$$

where *m* is the number of life stages. The age-specific survival rate (*l*_*x*_), the age-specific fecundity (*m*_*x*_), the finite rate of increase (*λ*), the intrinsic rate of increase (*r*), and the mean generation time (*T*) were calculated as previously described for the individually reared method.

For group reared method, the age-specific life expectancy (*e*_*x*_), i.e. the time that individuals of age *x* are expected to live^20^, is calculated as:

12 $${e}_{x}=\frac{\sum _{i=x}^{n}{l}_{i}}{{l}_{x}}$$

where *l*_*i*_ is the probability that an individual of age *0* will survive to age *i*. The reproductive value (*v*_*x*_) as the contribution of individuals of age *x* to future population^20^, is calculated as:

13 $${v}_{x}=\frac{{e}^{-r(x+1)}}{{l}_{x}}\sum _{i=x}^{\infty }{e}^{-r(i+1)}{l}_{i}{m}_{i}$$

To estimate the standard errors for population parameters using the bootstrap technique, the longevity data for each individual and daily fecundity of each female adult are needed. Because the age at death of each individual was recorded during the experimental period, the longevity and sex of each individual can be calculated. In addition, the information from the daily number of eggs laid by each living female was used to assign the mean fecundity to each female. This procedure does not affect the *l*_*x*_ and *m*_*x*_ because Chi^14^ validated the relationship between *R*_*0*_ and *F*. Therefore, this practice will not affect the population parameters. Due to the variable longevity of females, this practice can still reveal the variability of fecundity found in female adults. All data calculated for each individual were subjected to the bootstrap method with 40,000 resampling for estimating the standard errors of population parameters. Difference between treatments were then compared by using the paired bootstrap test^11, 27^.

## Electronic supplementary material

Dataset 1
