# Supplementary material for: Development of insect life tables: comparison of two demographic methods of Delia antiqua (Diptera: Anthomyiidae) on different hosts
Source: Sci Rep. 2017 Jul 6;7:4821. doi: 10.1038/s41598-017-05041-5 (PMC5500477; doi:10.1038/s41598-017-05041-5)
Supplement: Supplementary file 1 — Dataset 1 [file 41598_2017_5041_MOESM1_ESM.doc]

Development of insect life tables: comparison of two demographic methods of *Delia antiqua* (Diptera: Anthomyiidae) on different hosts

Shuo-ying Ning, Wenchao Zhang, Yan Sun, Ji-nian Feng^*^

*Key Laboratory of Plant Protection Resources and Pest Management of the Ministry of Education,* *College of Plant Protection，**Northwest A&F University,* *Yangling, Shaanxi, 712100, P. R. China;*

**The author for Corresponding: E-mail:* [*jinianf@nwsuaf.edu.cn*](mailto:jinianf@nwsuaf.edu.cn)

***P*-value of paired bootstrap test**

**Table S1 Paired bootstrap test of developmental time, adult longevity, fecundity, adult preoviposition period (APOP), total preoviposition period (TPOP), and oviposition days of *Delia antiqua* on different host plants of Individual reared data**

| Parameters | Stage | P value | | |
| --- | --- | --- | --- | --- |
|  |  | Onion to Scallion | Scallion to Garlic | Garlic to Onion |
| Developmental time (days) | Egg | 0.5478 | 0.0008 | 0.0000 |
|  | Larva | 0.9011 | 0.0000 | 0.0000 |
|  | Pupa | 0.9391 | 0.0000 | 0.0000 |
| Adult longevity (days) | Male | 0.3620 | 0.0000 | 0.0000 |
|  | Female | 0.8870 | 0.0000 | 0.0000 |
| APOP (days) |  | 0.0000 | 0.0000 | 0.0000 |
| TPOP (days) |  | 0.0437 | 0.0000 | 0.0000 |
| Fecundity (eggs) |  | 0.0000 | 0.0006 | 0.0000 |
| Oviposition days |  | 0.3417 | 0.0000 | 0.0000 |

The *P*- values which listed as 0.0000 indicate that the actual *P*-value is less than 0.0001 within the output confines of software 'TWOSEX-MSChart'.

**Table S2 Paired bootstrap test of net reproductive rate (*R_0_*), the intrinsic rate of increase (*r*), finite rate of increase (*λ*), and generation time (*T*) of *Delia antiqua* on different host plant of Individual reared data**

| Parameters | P value | | |
| --- | --- | --- | --- |
|  | Onion to Scallion | Scallion to Garlic | Garlic to Onion |
| *R_0_* | 0.0000 | 0.0000 | 0.0000 |
| *r* | 0.2126 | 0.0592 | 0.0003 |
| *λ* | 0.2159 | 0.0588 | 0.0032 |
| *T* | 0.0136 | 0.0000 | 0.0000 |

The *P*- values which listed as 0.0000 indicate that the actual *P*-value is less than 0.0001 within the output confines of software 'TWOSEX-MSChart'.

**Table S3 Paired bootstrap test of net reproductive rate (*R_0_*), the intrinsic rate of increase (*r*), finite rate of increase (*λ*), generation time (*T*) and fecundity of *D. antiqua* on different host plants of Group reared data**

| Parameters | P value | | |
| --- | --- | --- | --- |
|  | Onion to Scallion | Scallion to Garlic | Garlic to Onion |
| *R_0_* | 0.0040 | 0.0033 | 0.0072 |
| *r* | 0.2316 | 0.0976 | 0.0077 |
| *λ* | 0.2316 | 0.0972 | 0.0075 |
| *T* | 0.0003 | 0.0000 | 0.0000 |
| Fecundity (eggs) | 0.0011 | 0.0021 | 0.0000 |

The *P*- values which listed as 0.0000 indicate that the actual *P*-value is less than 0.0001 within the output confines of software 'TWOSEX-MSChart'.
